# Invertebrate Retinal Progenitors as Regenerative Models in a Microfluidic System

**DOI:** 10.3390/cells8101301

**Published:** 2019-10-22

**Authors:** Caroline D. Pena, Stephanie Zhang, Robert Majeska, Tadmiri Venkatesh, Maribel Vazquez

**Affiliations:** 1 Department of Biomedical Engineering, City College of New York, New York, NY 10031, USA; carolinedaniellapena@gmail.com (C.D.P.); rmajeska@ccny.cuny.edu (R.M.); 2 Department of Biomedical Engineering, The State University of New York at Binghamton, NY 13902, USA; szhan152@binghamton.edu; 3 Department of Biology, City College of New York, New York, NY 10031, USA; tvenkatesh@ccny.cuny.edu; 4 Department of Biomedical Engineering, Rutgers University, The State University of New Jersey, New Brunswick, NJ 08854, USA

**Keywords:** *Drosophila*, collective migration, neurons, glia, fibroblast growth factor

## Abstract

Regenerative retinal therapies have introduced progenitor cells to replace dysfunctional or injured neurons and regain visual function. While contemporary cell replacement therapies have delivered retinal progenitor cells (RPCs) within customized biomaterials to promote viability and enable transplantation, outcomes have been severely limited by the misdirected and/or insufficient migration of transplanted cells. RPCs must achieve appropriate spatial and functional positioning in host retina, collectively, to restore vision, whereas movement of clustered cells differs substantially from the single cell migration studied in classical chemotaxis models. Defining how RPCs interact with each other, neighboring cell types and surrounding extracellular matrixes are critical to our understanding of retinogenesis and the development of effective, cell-based approaches to retinal replacement. The current article describes a new bio-engineering approach to investigate the migratory responses of innate collections of RPCs upon extracellular substrates by combining microfluidics with the well-established invertebrate model of *Drosophila melanogaster*. Experiments utilized microfluidics to investigate how the composition, size, and adhesion of RPC clusters on defined extracellular substrates affected migration to exogenous chemotactic signaling. Results demonstrated that retinal cluster size and composition influenced RPC clustering upon extracellular substrates of concanavalin (Con-A), Laminin (LM), and poly-L-lysine (PLL), and that RPC cluster size greatly altered collective migratory responses to signaling from Fibroblast Growth Factor (FGF), a primary chemotactic agent in *Drosophila*. These results highlight the significance of examining collective cell-biomaterial interactions on bio-substrates of emerging biomaterials to aid directional migration of transplanted cells. Our approach further introduces the benefits of pairing genetically controlled models with experimentally controlled microenvironments to advance cell replacement therapies.

## 1. Introduction

Visual centers in the brain are activated when groups of progenitor cells interconnect to establish the highly organized, synaptic structure of neurosensory retina [1,2,3]. Critical to the formation of the retinal architecture are cell-cell interactions and cell-matrix interactions, which aid both our understanding of retinogenesis and the development of effective, cell-based approaches for regenerative medicine [3,4]. Migration is a central element of both development and regenerative processes because progenitors must move appropriately to align themselves with neighboring cell groups to establish tissue architecture [5,6]. In retina, both processes rely upon the collective migration of RPCs, i.e., movement of clustered cells as a group rather than as individual cells [3,5,6]. Such collective movements may differ substantially from the migration of individual cells studied in classical models of chemotaxis (reviewed in [7,8,9,10]), despite the same chemotactic stimuli driving locomotion. Specifically, individual cells chemotax independent of cell-cell adhesions, with a typical fibroblast locomotor cycle consisting of cellular protrusions and adhesion to the leading edge, development of contractile forces between the front and trailing edge, and then release of trailing adhesions due to the applied tension [11,12]. By contrast, cells moving collectively additionally depend intimately upon cell-cell interactions with one another and can achieve motion as organized cohorts of individually migrating cells, e.g., sheet migration [13,14] or as an interconnected clustered mass [6,15].

The complexity of progenitor cell movement presents distinct challenges to retinal regeneration because heterogeneous retinal clusters are comprised of cells of neuronal and glial lineages whose spatial organization, and their effects on RPC migration, remain incompletely understood [5,16]. While contemporary cell replacement strategies have utilized a growing number of transplantable biomaterials to aid viability of transplanted cells [17,18,19], inadequate and/or misdirected cell migration into damaged retina has been cited as a primary factor in the inability to achieve synaptic integration and restore vision [20,21,22,23]. Bio-engineering techniques and approaches with which to understand how the migratory responses of transplanted RPCs are mediated by their interactions with one another, soluble chemotactic stimuli, and extracellular substrate(s) will, thereby, greatly enrich retinal transplantation strategies.

The precisely-controlled and defined environments created using microfluidic systems have heralded tremendous advances in biology and regenerative medicine over the past decade [24,25,26,27], not only in technological development of single cells and high throughput microdevices [28,29], but additionally in the emergence of microfluidically-manipulated biomaterials for tissue grafts and organs on a chip [30,31,32]. Numerous groups, including our own, have developed microfluidic systems for precise analyses of neural migratory responses within defined concentration gradients of chemotactic stimuli using a variety of experimentally determined substrates and surfaces [33,34,35,36,37,38,39]. Regenerative medicine has further pioneered the growth of micro and nanotechnologies in tandem with the isolation/application of stem-like cells for cell replacement therapy [40,41,42]. Moreover, its recent integration with biomaterials has empowered the study of cell processes and organization upon bioengineered substrates and surfaces used for tissue engineered cellular grafts [43,44,45] and emerging organoid models [46,47,48]. Despite these exciting advances, however, the extent to which outcomes of retinal transplantation are mediated by collective migratory behaviors within and upon extracellular substrates has been incompletely explored.

The current article describes a new bio-engineering approach to investigate the migratory responses of innate collections of individual RPCs and collective RPC clusters by combining microfluidics with the well-established invertebrate model of *Drosophila melanogaster*. The genetic flexibility of *Drosophila* models has made the fly eye a seminal model in development and has demonstrated that signaling pathways governing both phototransduction and retinal architecture are well-conserved among species (Reviewed in [49,50,51]). Integration of invertebrate genetic models used to elucidate cell-cell and cell-substrate signaling critical to both development and regenerative strategies will greatly advance emerging biomaterials to aid retinal transplantation.

Previous work from our group [52] illustrated that primary RPCs isolated from *Drosophila* migrated as clusters within signaling gradient fields, with little to no directional motility observed from singleton cells. The current project applied microfluidics to further investigate how cluster composition, size, and adhesion on defined extracellular substrates affected RPC migration to exogenous chemotactic signaling. Experiments extracted RPCs from primary eye-brain complexes of *Drosophila melanogaster* and quantified differences in cell attachment, cluster size, and ratios of adhered RPC clusters to individual cells upon substrate coatings of concanavalin (Con-A), Laminin (LM), and poly-L-lysine (PLL). These matrixes were chosen because of their significance to the development of contemporary biomaterials in the visual system. The lectin, ConA, recognizes cell surface carbohydrates common across species and has been used extensively as an adhesive substrate for cells within the visual system [53,54]. PLL is a positively charged polymer that promotes strong adhesion of virtually all cell types based solely on their negative surface charge [54]. Laminin is a component of basement membranes found at interfaces between tissues derived from distinct developmental origins (e.g., epidermis and dermis of skin, vascular endothelium and surrounding vessel layers) where cell migration during development frequently occurs. Laminin has also been commonly used as a substrate in development of retinal organoids [55] and transplantable retinal biomaterials [33]. Results demonstrated that retinal cluster size and composition influenced RPC responses to signaling from Fibroblast Growth Factor (FGF), a primary chemotactic agent in Drosophila (Reviewed in [56,57]). Surprisingly, retinal clusters of different sizes migrated preferentially along different FGF signaling fields, with larger clusters illustrating larger directionality and migration distances. These results highlight measurable differences between individual and collective RPC responses on transplantable biomaterial substrates. Further, our bio-engineering approach leveraged genetically-controlled models with experimentally-controlled microenvironments to enhance development of retinal biomaterials via study of collective RPC adhesion and migration.

## 2. Materials and Methods

### 2.1. Drosophila Fly Stocks

Experiments utilized the GAL4-UAS system [58], in which glial and neuronal precursors express green and red fluorescent protein (GFP, RFP), respectively. *Drosophila melanogaster* stocks of UAS-GFP (CS: Repo) and UAS-mCD8-GFP; elav GAL4 were used because the Elav (neurons) and Repo (Glia) markers are the only markers to specifically stain cells in the developing retinal ganglion [59]. We note that less than 5% of the total cell sample did not stain for either neurons or glia. Flies were maintained on standard corn meal agar medium and kept at 25 °C. Stocks were transferred once a week to maintain lines of larvae mixed from the two strains.

### 2.2. Dissection, Dissociation and Cell Culture

**Figure 1:**
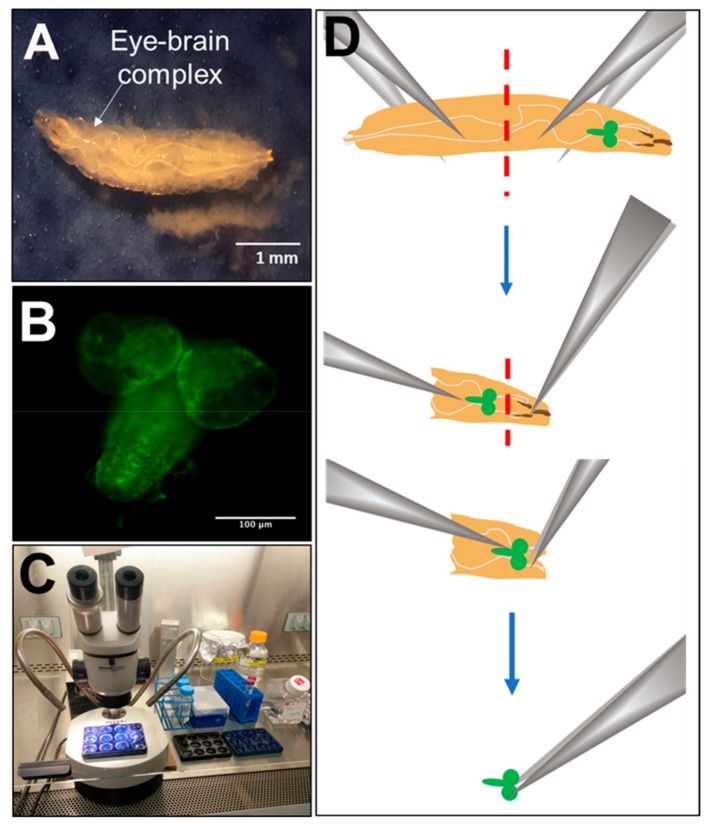
*Drosophila melanogaster* model. (**A**) Image of third instar larva. (**B**) Dissected eye-brain complex with GFP+ glia (Scale bar: 100 µm). (**C**) Dissection arrangement via microscope within a laminar flow hood. (**D**) Schematic of key steps in the dissection process, where third instar larvae are segmented using tweezers, and mouth hooks with excess tissue are removed to isolate eye-brain complexes (green cartoon).

Eye-brain complexes were isolated from third instar larvae using methods based on established studies [60,61,62] and performed in a laminar flow hood (Figure 1). A minimum of 15–20 eye-brain complexes were dissected using stainless steel #5 tweezers in phosphate buffered saline (PBS) and washed once with Schneider’s medium (Thermo Fisher Scientific, Waltham, MA, USA) supplemented in 10% (*v/v*) heat inactivated fetal bovine serum (FBS) and 1% (*v*/*v*) penicillin streptomycin (Gibco, Grand Island, NY, USA). Note that Schneider’s medium was made the same day as dissection and dissociation. Complexes were kept in 40 μL of PBS on ice until 15–20 complexes were gathered. Complexes were digested in a 1 mL volume of 0.5 mg/mL collagenase (Gibco, Grand Island, NY, USA) at 25 °C for 1 h, centrifuged at 2000 rpm for 5 min and washed twice by re-suspending in 1 mL of supplemented Schneider’s medium. Tissues were further mechanically disassociated by pipetting in 150 μL of supplemented Schneider’s medium (10 μL per brain) and straining through a cell strainer of 40 μm diameter pore size. Resultant cell suspensions were maintained at 25 °C (Barnstead Labline L-C incubator, Thermo Fisher Scientific). An immortalized S2 *Drosophila* cell line derived from embryos [63] was also cultured under identical conditions as a control to verify an adequate growth environment in vitro. Note that standard cell culture temperature for *Drosophila* is between 25 °C and 28 °C [49] in contrast to the 37 °C of conventional mammalian cell culture [64].

### 2.3. Immunocytochemical Assessment of Neuronal and Glial Marker Expression

After dissection and dissociation, neural cells were incubated overnight in Petri dishes in a 25 °C incubator. Glass slides were coated with 15 µg/mL of Concanavalin A (eBioscience, Carlsbad, CA, USA) and briefly heated on a hot plate at 100 °C. The cell suspension was centrifuged at 2000 RPM for 8 min; 110 µL was discarded and cells were re-suspended in the remaining 40 µL. The cell suspension was then placed on the coated glass slide for 30 min at room temperature (25 °C) to facilitate cell attachment. The supernatant was removed, and the cells were fixed in 40 µL formalin (buffered 4% formaldehyde) (Sigma-Aldrich, St. Louis, MO, USA) for 15 min. The formalin was removed, and fixed cells were washed 3× with PBST (0.1% Triton X-100) (Sigma-Aldrich). Primary glia-specific antibodies 8D12 anti-Repo (Developmental Studies Hybridoma Bank, Iowa City, IA, USA) and neuron-specific Rat-Elav-7E8A10 anti-Elav (Developmental Studies Hybridoma Bank) were diluted in PBST and added to fixed cells. The slides were incubated overnight at 4 °C. Unbound antibody was removed by washing the slide 3× for 2 min and 2× for 10 min with PBST. Secondary antibodies Alexa Fluor 488 goat anti-mouse IgG (Invitrogen, Carlsbad, CA, USA), and Alexa Fluor 594 goat anti-rate IgG (Invitrogen) were diluted in PBST and added to the slide. The slide was incubated for 2 h at room temperature and then washed 3× for 2 min and 3× for 10 min. All supernatant was removed from slides and mounted with ProLong Diamond Antifade Mountant (Invitrogen).

### 2.4. Substrate Influence on Cell Survival and Morphology

Three extracellular substrates were tested for their ability to support cell viability and to modulate the morphology of individual cells and cell clusters. Glass bottom 47 mm diameter Petri dishes (MatTek, Ashland, MA, USA) were coated with 300 µL of 100 µg/mL Poly-L-lysine (PLL, Sigma-Aldrich), 15 µg/mL Concanavalin A (Con-A, eBioscience) or 80 µg/mL Laminin (LM; Gibco, Grand Island, NY, USA) at room temperature (25 °C) for 1 h. Uncoated dishes served as controls. The supernatant was then removed, and the dishes were washed 3× with PBS. All liquid was removed from the dish and placed in a 25 °C incubator overnight. Cell cultures were maintained in a 25 °C incubator and fresh Schneider’s medium was added to cell cultures after 24 h. Cells were assessed for morphology and viability at 0, 24 and 48 h. Brightfield images of cell cultures were taken to assess morphology of individual cells and RPC clusters. Viable cells on each substrate were tested after 24 h and 48 h using the Colorimetric Cell Viability Kit III XTT (Invitrogen). Potential reductions in cell viability over time were assessed by comparing XTT absorbances with values obtained from assays of samples of newly-dissected cells (n > 15 eye-brain complexes, isolated as described). All absorbance values were normalized against those on uncoated Petri dishes.

### 2.5. The μLane Migration Assay

**Figure 2:**
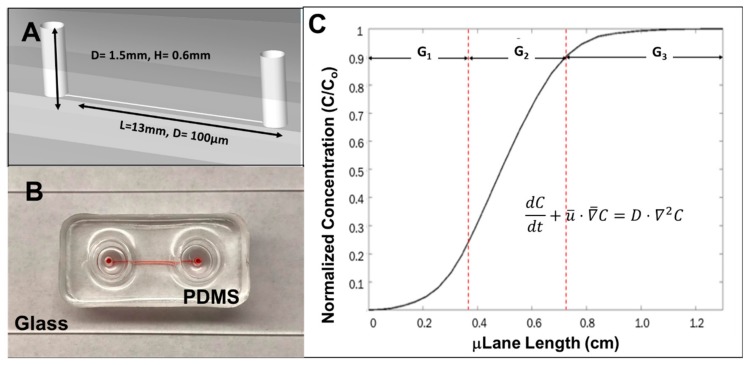
Overview of the µLane system. (**A**) Schematic of microfluidic system comprised of two volumetric reservoirs connected by a 200-micron-diameter channel. (**B**) Image of fabricated device loaded with red dye. (**C**) The distribution of FGF concentration achieved within the µLane, normalized to the input concentration, Co. Transport within the µLane is defined by the convective-diffusion equation shown, where areas of mathematically-distinct changes in concentration gradients are defined along different lengths of the microchannel as marked: G_1_, G_2_ and G_3_.

The μLane system has been described previously by our group [65] and was used to analyze chemotactic processes of cells derived from a variety of animal models [23,66,67,68]. As shown in Figure 2, the μLane system used in this study consists of a large volume source reservoir (0.6 mm diameter, 0.6 mm depth) connected to another large volume sink reservoir (0.6 mm diameter, 0.6 mm depth) by a microchannel of 100 μm diameter and 1.2 cm length. This geometry is created by micromolding of layers of poly-dimethylsiloxane (PDMS), and this elastomer is later ozone-bonded to a transparent glass side or coverslip to create a closed microfluidic system. Transport within the adjoining microchannel is defined by the convection-diffusion shown in Equation (1), used to quantitatively determine the concentration profile within the system:

(1) $$\frac{dC}{dt}+\bar{u}\cdot \bar{\nabla }C=D\cdot \nabla^{2}C$$

where C represents concentration in g/mL, t is time measured in s, u is bulk velocity in m/s and D represents molecular diffusivity in m^2^/s. The concentration gradient of FGF-8 (Invitrogen) within the µLane system was established by loading a 100 ng/mL concentration of reagent into the source reservoir (reference point = 0 cm) while the remaining system was filled with media. Transport of FGF from high concentration in the source reservoir to low concentration in the sink reservoir established a non-linear concentration gradient profile within the microchannel. All inner surfaces of the system were coated with selected bio-substrates prior to migration testing.

In brief, based on the distributions of solute concentration within the µLane system as a function of time, it was possible to establish both the concentration of solute, C, and the concentration gradient, G (i.e., change in concentration over distance), of each location at each time after addition of the test solute (FGF). The movement of individual cells at a given time and location could thus be related to both solute concentration and gradient. Three distinct regions of the µLane system were designated as exhibiting different solute concentration gradients for ease of analysis: G_1_, G_2_ and G_3_. These distinct areas were selected because they represented regions of the highest mathematical change in reagent concentration over length, i.e., gradient. As shown in Figure 2, the area of G_1_ is present within the first section of channel length, L_1_, from 0 cm to 0.35 cm and represents normalized percent change in concentration of 25%. This equates to an average gradient field of G_1_ = 7.15 ng/mL per mm of channel and additionally corresponds to the lowest concentration range between C = 0 and 25 ng/mL. The area of G_2_ is defined at the channel mid-region between L_2_ = 0.35 cm to 0.75 cm of the µLane system and denotes a concentration change of 65%. This results in an average gradient field of G_2_ = 16.25 ng/mL per mm of channel and a mid-concentration range between 25 ng/mL and 90 ng/mL. The last gradient region, G_3_, is established between L_3_ = 0.75 cm to 1.3 cm of the µLane system and denotes a normalized concentration change of 10% for an average gradient field of G_3_ = 1.63 ng/mL per mm of channel with the highest concentration range upwards of 90 ng/mL. Cells were seeded into the μLane cell reservoir while FGF was added to the device source well. Control experiments utilized Schneider’s media only, without additional FGF. A transport-driven gradient was developed within the adjoining microchannel, and cell migration was recorded every hour within different G_1_, G_2_ and G_3_ regions for a total of 8 h.

### 2.6. Microscopy and Imaging

A Nikon Eclipse TE2000 inverted microscope (Morell Instruments, NY, USA) with a 20× objective was used in conjunction with the NIS Elements Imaging Software to gather fluorescent images of larvae, eye-brain complexes, fixed and stained cells. Confocal images of fixed and stained cells were captured using a Zeiss LSM 800 (Zeiss, Jena, Germany) with Airyscan under 40× and 63× oil objective. An argon laser at 488 nm and 594 nm and was used to excite immunostained glial and neuronal progenitors, respectively.

Brightfield images of cells on ECM-coated substrates were captured at 20× and 40× magnification using a Nikon Eclipse TE300. Brightfield images of μLane devices were gathered at 20× every 1 h for 8 h within 5 specified regions of the defined regions of each of the G_1_, G_2_ and G_3_ gradients.

### 2.7. Data Analysis

Data were analyzed using ImageJ (NIH). The total numbers of cells and cells per brain were calculated via cell counting using a hemocytometer and Trypan Blue. An average of 10 larval samples were selected to determine a mean value of area for both single cells and clusters. The ratio of cells of neuronal lineage to total cells, R_N_, was analyzed via fluorescent optical imaging using the cell counter plugin, defined in Equation (2):

(2) $$R_{N}=\frac{\mathrm{Total}\;\mathrm{number}\;\mathrm{of}\;\mathrm{cells}\;\mathrm{with}\;\mathrm{neuronal}\;\mathrm{lineage}}{\mathrm{Total}\;\mathrm{number}\;\mathrm{of}\;\mathrm{cells}\;}$$

A total of 1993 single cells and 224 RPC clusters were examined for 3 independent experiments. The morphology of the adhered individual cells and clusters was analyzed using cell shape index (CSI) and the average surface area, respectively. CSI has been previously utilized by our group and others [59,60,61] as a dimensionless parameter to quantitatively represent cell asymmetry, as defined in Equation (3):

(3) $$\mathrm{CSI}=\;\frac{4{\pi A}_{s}}{p^{2}}$$

where A_s_ represents cell surface area and P denotes cell perimeter. The average surface areas of clusters that adhered to substrate surfaces after 24 h were measured using ImageJ. The ratio of clusters to individual cells, R_RC–IC_, was examined both in freshly isolated cell suspensions and after adherence to coated plates using Equation (4):

(4) $$R_{\mathrm{RC}-\mathrm{IC}}=\frac{\mathrm{Total}\;\mathrm{number}\;\mathrm{of}\;\mathrm{RPC}\;\mathrm{clusters}}{\mathrm{Total}\;\mathrm{number}\;\mathrm{of}\;\sin\mathrm{gle}\;\mathrm{cells}}$$

Characteristics and behavior of both single cells and clusters in the μLane system were evaluated in all three regions of the concentration gradient, G_1_, G_2_, and G_3_. Cells and clusters were tracked individually on ImageJ using the Manual Tracking plugin. Retinal clusters were tracked using the center of mass. Motile cell trajectories were graphed using normalized X and Y points for the nine time points recorded via time-lapsed cell migratory studies [62,63,64]. Representative trajectories were chosen to display the average movement of single cells, small clusters and large clusters in each gradient field. The average total path length, L_p_, or sum of the distance travelled, was determined using Equation (5) and Equation (6):

(5) $$\;l=\;\sqrt{\left|{\left(X_{2}-X_{1}\right)}^{2}-{\left(Y_{2}-Y_{1}\right)}^{2}\right|}$$

(6) $${\;L}_{P}=\;\overset{n}{\underset{i=1}{\sum }}l_{i}\;$$

where X and Y represent spatial positions of motile cells within the μLane system at two consecutive time points, 1 and 2. The total path length, L_P_, was then calculated by summing the path lengths over the entire trajectory of single cells and clusters in each gradient region. The average path length was plotted for single cells and clusters of small and large size.

Directional cell migration was defined by the chemotactic index, CI, previously used by our group and by others as shown in Equation (7):

(7) $$\mathrm{CI}=\;\frac{x}{L}\;$$

where x is the distance moved towards the gradient, and LP is the path length from Equation (6). In this study, values of CI approach 1 as cells move in the direction of increasing gradient and become negative when cells migrate away from the gradient. The chemotactic index was calculated for single cells and small and large clusters within the gradient regions of the µLane system, G_1_–G_3_.

ImageJ was used to analyze neuronal and glial marker expression, cell morphology on extracellular substrates, and cell migration within the µLane system. Numbers of cells that expressed the neuronal and/or glial marker were determined using the Cell Counter feature of the Analyze plugin. The cell shape index (CSI) and surface area of cells were determined using the Analyze Particles function, which returns circularity and area values. Cell migration was analyzed using the Manual Tracking feature of the Tracking plugin, which returns spatial positions (x, y) for individual cells and clusters over time.

### 2.8. Statistical Tests

Statistical analysis was performed using the Statistics and Machine Learning Toolbox of Matlab (Mathworks, Natick, MA). One-way analysis of variance (ANOVA) was used for testing variance when using the independent variable of interest, i.e., FGF concentration gradient. A 95% confidence interval and a post-hoc test (Tukey) for comparing multiple samples were used. The ANOVA confirmed statistical differences amongst control and experimental groups while the post-hoc (Tukey) test determined differences amongst experimental groups. Statistical analyses were performed amongst the different gradient regions of each experimental group and between the experimental groups themselves to determine how changes in concentration gradient influenced the distances travelled measured via path length, L_P_, and directional movement was assessed via the chemotactic index, CI. Calculated *p* values <0.05 were considered statistically significant and were represented with a single asterisk in all figures, while a double asterisk was used to denote lower *p* values < 0.01. In addition, a sample t-test (α = 0.01) was performed to determine the distribution of x positions, the direction in which the chemotactic gradient is not distributed, of motile single cells and clusters analyzed. Normalized distributions of data points were assessed via skewness and kurtosis [69]. Skewness, measure of symmetry, and kurtosis, measure of the tails of the distribution, provide measures of shape of the data. An ideal, normally distributed data set exhibits skewness and excess kurtosis of 0 with acceptable ranges between −2 to +2 [70]. Lastly, a Jarque-Bera test (α = 0.01) [71] was performed to identify statistically significant variation from normal distributions.

## 3. Results

This study examined how chemical cues from a controlled signaling microenvironment influenced the independent and collective chemotactic behavior of heterogeneous populations of retinal progenitor cells (RPCs). All tests were performed using primary RPCs dissected from eye-brain complexes of third instar larvae *Drosophila*. We note that RPCs in the larval retinal ganglion are neuroblasts capable of differentiating into neurons or glia [59].

### 3.1. Neuron:Glia Distribution

**Figure 3:**
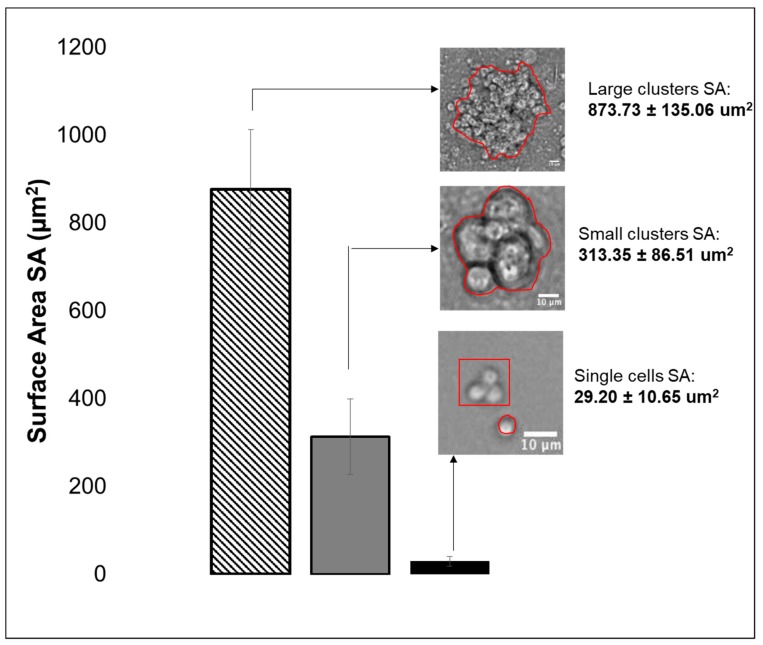
Distributions of collective RPC clusters and individual cells post-dissection. Average surface areas of individually adhered cells and adhered RPC clusters of small and large size. A representative small cluster of approximately 3 cells is shown alongside a singleton cell to demonstrate consistency with the size and shape of single cells. Error bars the denote standard deviation.

**Figure 4:**
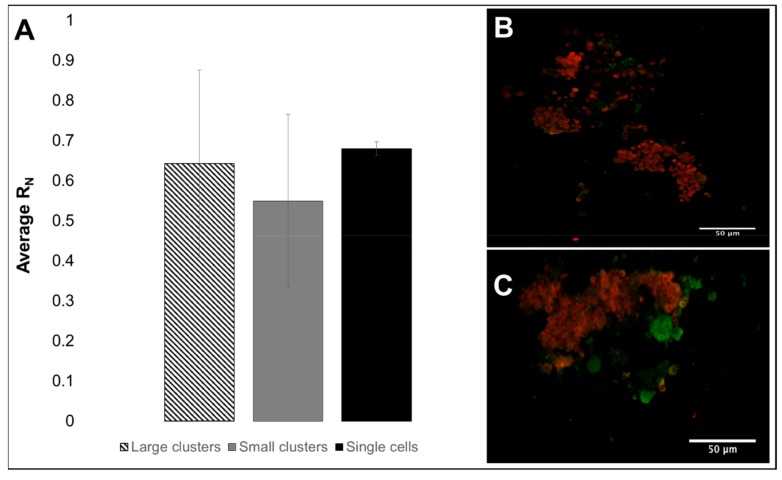
Average ratio of neuronal progenitors to total cells, R_N_, in third instar larvae. (**A**) Average ratio of neuronal cells to total cells (R_N_) in large clusters, small clusters and single cells, obtained from immunostaining. Error bars denote the standard deviation. (**B**,**C**) Representative confocal images of RFP+ neurons and GFP+ glia at mid-plane.

Experiments first estimated the total number of cells per eye-brain complex harvested from the third larval stage to utilize a consistent cell density per complex for in vitro testing. Dissections of n= 15 and n= 30 eye-brain complexes were performed in triplicate, and cells were counted via hemocytometer to denote an average of 104 cells per brain, as per Table 1. Cells were then plated onto Petri dishes and examined for aggregation into retinal clusters after 6 h, as shown in Figure 3. Three populations of cells were observed based on the size of clusters formed: (a) Individual cells (IC) of 5–6 microns in average diameter; (b) Small retinal clusters (SC), defined as clusters of 5 to 15 cells; and (c) Large retinal clusters (LC) comprising more than 15 cells. Average cluster size was assessed by measuring the surface area of substrate, SA, occupied by each cell or cluster. The average size of individually adhered cell groups was ^IC^SA= 29.20 ± 10.65 µm^2^, while small clusters exhibited an average surface area of ^SC^SA 313.35 ± 167.51 µm^2^, and large clusters an average surface area of ^LC^SA 573.73 ± 135.06 µm^2^. The ratio of neuronal to total cells, R_N_, determined by immunostaining, was ^IC^R_N_ = 0.68 ± 0.017 for single cells as shown in Figure 4. Similar ratios, based on the relative area of immunostaining in small clusters, ^SC^R_N_ = 0.55 ± 0.22, and large clusters, ^LC^R_N_ =0.64 ± 0.23, were not significantly different from those in individual cells (*p* > 0.05).

### 3.2. Viability, Adhesion and Cluster Formation upon Different Substrates

**Figure 5:**
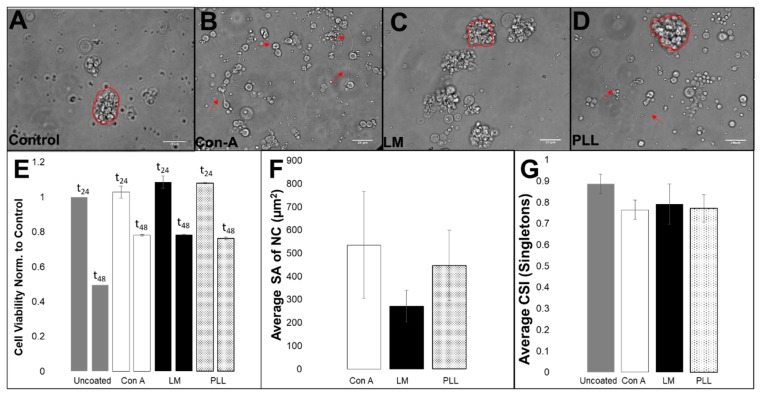
Viability and adhesion of disassociated cells upon extracellular substrates. Primary RPCs upon (**A**) uncoated Petri dish, (**B**) Concanavalin A (Con-A) with neurite extensions highlighted by arrows, (**C**) Laminin (LM) with outlined RPC clusters and (**D**) Poly-L-Lysine (PLL) with both RPC clusters and neurite extensions highlighted. (**E**) Normalized cell viability at 24 h and 48 h time points. (**F**) Average RPC cluster surface area (RC SA) on Con-A, LM and PLL. (**G**) Average values of cell shape index (CSI) measured at 24 h time point for cells adhered on Con-A, LM and PLL. Error bars denote standard deviation. (Scale bar = 20 µm for all images.). Statistical significance (*p* < 0.05) against control is denoted by *.

Adhesion of RPC upon Poly-L-lysine (PLL), concanavalin A (Con-A) and laminin (LM) surfaces was assessed by viable cell staining with the metabolic dye XTT to determine whether these substrates could promote the survival of cells derived from the third larval instar over that on standard cell culture plastic surfaces. At 24 h after plating, XTT staining indicated comparable numbers of viable cells on all substrates. However, numbers declined by 50% in control plates at 48 h after plating but by only 20% on other substrates, as per Figure 5.

Differences in the proportion of retinal clusters (RC, all sizes) to individual cells, R_RC-IC_, average cluster surface area, SA, and the ratio of small to large clusters, R_CL_ were assessed for each substrate. As seen in Figure 5, seeded RPC illustrated different mixtures of adhered clusters and single cells upon PLL, Con-A and LM. Cells adhered predominantly as single cells upon Con-A with an adhesion ratio of ^ConA^R_RC–IC_ = 1/5. By contrast, RPC adhered to PLL with a 1:2 ratio of clusters to individual cells, ^PLL^R_RC–IC_ = 1/2, and to LM with the largest clustering of ^LM^R_RC–IC_ = 2/1. Notably, the average surface area, SA, of adhered clusters was opposite to the measured preference of cluster formation with values of ^LM^SA = 271.6 ± 69.12 µm^2^ upon LM, ^PLL^SA = 447.6 ± 151.2 μm^2^ on PLL and ^ConA^SA = 535.8 ± 232.1 μm^2^ upon Con-A. Further, the clustering ratio of small to large clusters, R_CL_, was approximately ^ConA^R_CL_ = 1:1 on Con-A, ^LM^R_CL_ = 1:15 on LM and ^PLL^R_CL_ = 10:1 on PLL. Lastly, the cell shape index (CSI) of individually adhered cells was the greatest (i.e., most rounded) on uncoated control plates, with an average value of ^CNTRL^CSI=0.89 ± 0.05, and lower on all other substrates with ^PLL^CSI = 0.77 ± 0.05 on PLL, ^LM^CSI = 0.79 ± 0.09 and ^ConA^CSI = 0.76 ± 0.06. No statistical differences were measured across substrates (*p* > 0.05).

### 3.3. RPC Chemotactic Migration

**Figure 6:**
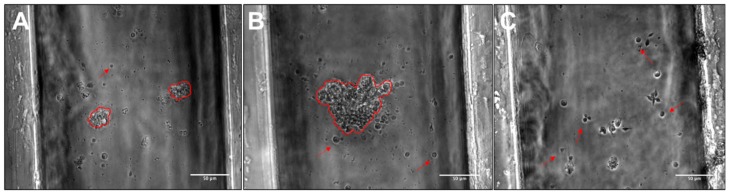
Adhesion of retinal progenitor groups within the µLane system. The three populations of cells observed in suspension and on ECM-treated plates were also seen within the µLane system. Representative images of (**A**) Small RPC clusters and individual cells, (**B**) Large RPC clusters and individual cells and (**C**) Individual cells within the µLane system (Scale bar = 50 µm).

**Figure 7:**
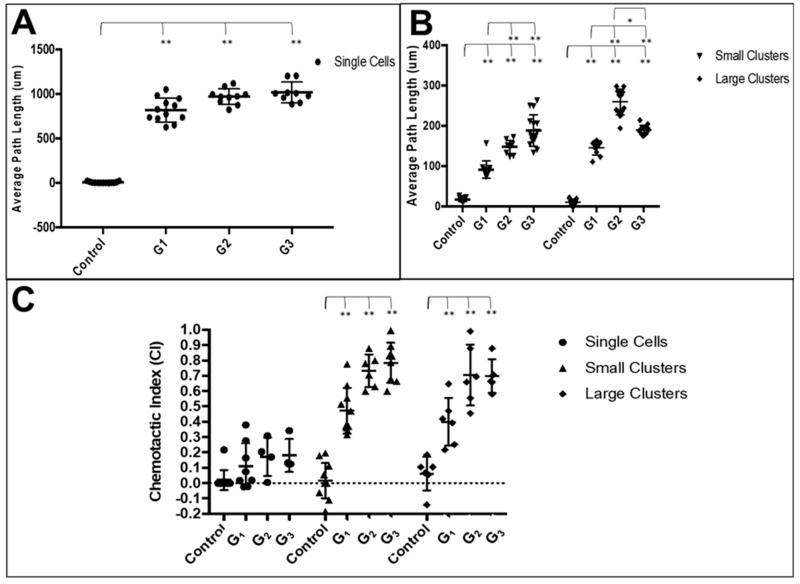
Bee swarm plots of average path length and chemotactic index. (**A**) Average path length of single cells in control and gradient fields, G_1_, G_2_ and G_3._ Statistical significance ** *p* < 0.01 between control groups and experimental groups. (**B**) Average path length of small and large RPC clusters in control (i.e., Schneider’s medium only) and gradient fields, G_1_, G_2_ and G_3_, generated within µLane. Statistical significance ** *p* < 0.01 between control and experimental groups. Statistical significance * *p* < 0.05 between medium and high gradient fields in large clusters. (**C**) Chemotactic index, CI, of single cells, small and large RPC clusters in control and gradient fields, G_1_, G_2_ and G_3._ No statistical significance amongst groups for single cells. Statistical significance ** *p* < 0.01 between control groups and experimental groups for RPC clusters.

The final set of experiments evaluated the directional migration of RPCs using the µLane system to generate controlled concentration gradient fields of FGF, as shown in Figure 2. The well-defined process of gradient development enabled analysis of individual gradient fields, G_1_-G_3_, along different lengths of the device, L_1_-L_3_, such that both the concentration, C, and gradient of FGF were known at each site. Figure 6 illustrates that RPC adhered as both clusters and individual cells within the device, as was previously observed in mass cell culture. The average path lengths and chemotactic index (CI) of individual cells and small and large clusters of RPCs within the gradient fields of the µLane system are represented as Bee Swarm Plots in Figure 7.

Data illustrate that the average path length of motile individual cells within G_1_ gradient fields was ^G1^L_IC_ = 819.4 µm. The average path length in G_2_ gradient fields was ^G2^L_IC_ = 987.9 µm and ^G3^L_IC_ = 1018.6 µm in G_3_ gradient fields. All experimental groups migrated farther than controls (*p* < 0.01) in each gradient field (G_1_, G_2_, G_3_), but no statistical difference was measured among motile groups in the three fields. In addition, the directionality of single cell movement was evaluated using the chemotactic index, CI, defined in Equation 3. As seen, average CI values for single cells in the different gradient fields were low, indicating no directionality of movement. Measured CI values per gradient field were ^G1^CI_IC_ = 0.16 ± 0.21, ^G2^CI_IC_ = 0.24 ± 0.19 and ^G3^CI_IC_ = 0.26 ± 0.19, with no statistical significance between groups (*p* > 0.05).

In contrast to single cell movement, small clusters (SC) displayed much smaller average path lengths of ^G1^L_SC_ = 98.6 μm in G_1_ gradient fields, ^G2^L_SC_ = 160.3 μm in G_2_ gradient fields and ^G3^L_SC_ = 188.2 μm in G_3_ gradient fields. However, the migration of clusters was directional, with larger average CI values of ^G1^CI_SC_ = 0.51 ± 0.15, ^G2^CI_SC_ = 0.73 ± 0.11 and ^G3^CI_SC_ = 0.80 ± 0.16 in respective gradient fields. Again, all experimental groups migrated further than control cells (*p* < 0.01) and values measured at G_2_ were significant relative to G_1_ and G_3_. Large clusters (LC) illustrated similar average path lengths of ^G1^L_LC_ = 144.6 μm, ^G2^L_LC_ = 258.7 μm and ^G3^L_LC_ = 189.8 μm in respective gradient fields. The average CI values of large clusters were ^G1^CI_LC_ = 0.50 ± 0.16, ^G2^CI_LC_ = 0.70 ± 0.20 and ^G3^CI_LC_ = 0.73 ± 0.10. Statistical significance (*p* < 0.01) was measured between the control and each experimental group for both average path length and CI.

The data represent preferential movement in the y-direction of changing concentration gradients, with minimal movement in the x-direction for all motile single cells and clustered groups. Statistical analysis indicated a normal distribution of x positions about the channel centerline (x = 0) for all cases, with statistical values of skewness and kurtosis near 0 for all motile groups. These results illustrate no statistical bias of motion in the x-direction caused by the device or experimental setup itself. Lastly, Jarque-Bera tests (α = 0.01) yielded p-values that demonstrated data for single cells, small clusters and large clusters did not significantly differ from normally distributed data.

## 4. Discussion

Contemporary regenerative therapies have begun to introduce retinal progenitor cells (RPCs) to replace dysfunctional or injured cells using customized biomaterials that enhance viability and function during transplantation [17,19]. However, regenerative outcomes have been severely limited by the misdirected and/or insufficient migration of transplanted RPCs [20,21], whose collective migration is needed to restore vision via appropriate spatial and functional positioning [72,73]. This limitation is due, in large part, to limited understanding of the collective migratory processes of heterogeneous clusters of neuronal and glial progenitors. Our study used controlled microenvironments to examine how collective chemotactic processes are influenced by the size and lineage composition of RPC clusters, and their adhesion upon defined extracellular substrates. In tandem, we here explored a new bio-engineering approach to leverage extensive retinogenesis data by using primary invertebrate cells isolated from *Drosophila melanogaster*.

### 4.1. Invertebrate RPC Models In Vitro

*Drosophila* is a seminal developmental model that has enabled transformative genetic advances in the study of signaling pathways regulating cellular retinal structure and coordinated phototransduction processes across species [74,75,76]. *Drosophila* RPCs can be genetically manipulated, exactly, to better regulate and/or understand the cell-cell and cell-matrix signaling needed for their collective RPC migration [73]. This invertebrate model, thereby, provides unique opportunities for development of biomaterials used in regenerative therapies for inherited diseases and retinal disorders more broadly [77]. In vitro study of the collective behaviors of *Drosophila* progenitors has been surprisingly scarce [52,78,79,80,81], largely because cells extracted from developing organisms are notoriously difficult to maintain in vitro: The average viability of cells isolated from *Drosophila* visual system has been reported to be 12% after 24 h [46,82]. We here report achieving cell viability approaching 80% after 48 h in vitro (Figure 5), presumably by using sterility protocols of mammalian cell culture in combination with specific substrate-coated surfaces (Figure 1). Such substantial increases in cell survival facilitated reproducible in vitro testing for 8–10 h, post-isolation, and will enable future study using cells from genetic disease models that are promising candidates for regenerative therapy.

### 4.2. Ratio of Neuronal:Glial Progenitors 

In vitro experiments first measured cell lineage composition and aggregate size of isolated RPCs. The ratio of neurons to total cells, R_N_, is highly significant to transplantable biomaterials because it impacts the cell-to-cell communication required to maintain and regulate collective behavior [1,2,83]. Surprisingly, the ratio of neurons to glia at the third larval instar has been largely unmeasured and/or unreported despite its wide applicability to studies of retinogenesis [75]. Rather, R_N_ has been estimated to be closest to that of adult flies, given its later stage of development. These values have been reported to be as high as 1:1 (R_N_ = 0.50) in *Drosophila* embryos and to decrease to approximately 1:10 (R_N_ = 0.10) in adult flies [49,72]. By contrast, our project measured an average R_N_ value of 1:2.5 (R_N_ = 0.62) using both hemocytometer cell counts and confocal microscopy of fluorescent cells (Figure 4). We note that cell isolation procedures used in this study produced a minute population of non-neural cells (<5% total), which had no impact on the calculation of the R_N_ ratio. Significantly, values of R_N_ averaged approximately 0.62 across both individual RPCs and retinal clusters of different sizes. Furthermore, this ratio of neuronal to glial progenitors was observed within motile clusters in μLane, reinforcing the persistence of the R_N_ parameter. These newly measured data provide an important, preliminary reference point in the study of RPC migration that highlights the need to identify lineage variance in cells introduced for regenerative therapy. However, further study is needed to examine the underlying mechanisms for this particular ratio of neuronal to glial progenitors at late stages of retinal development. Future tests will also use qPCR to quantitate these differences.

### 4.3. Collective RPC Interactions with Extracellular Substrates

As cell-based therapies increasingly rely upon transplantation of cells in combination with extracellular matrix-based scaffolds [45,84,85], our second set of experiments examined RPC interactions with substrates commonly used for this purpose. ConA is a lectin molecule well studied in the retinal tissue of both invertebrates and vertebrates [53] and used as an adhesive substrate for retinal cells in vitro [86]. Most recently, ConA was used to induce RPC migration from retinal grafts [87] as well as to suppress proliferative vitreoretinopathy in rats [88,89]. Both ConA and PLL have been used as in vitro substrates to examine survival of retinal neurons from amphibians, avians, invertebrates and mammals [86,90]. Further, PLL has most recently been studied as a substrate for drug delivery to the retina [91,92], for retinal transplantation [93], and as coatings for retinal implants used to enhance and direct regrowth of ganglion axons [54,94]. Lastly, Laminin was selected for study because it is ubiquitous in the visual system as well as critical for retinal lamination [95]. Recent projects have additionally used Laminin in development of retinal organoids [55], transplantable biomaterials for retinal replacement [33,96,97], and as part of substrates used to model retinal disease [98,99,100,101].

Measurements of average cluster size suggest that RPCs demonstrate innate preferences for the size of RPC collectives, as two thirds of extracted cells self-aggregated into clusters with a larger surface area representative of 15–20 cells (Figure 3). Cell-biomaterial interactions are, thereby, highly significant as different substrates can influence inherent cell clustering and/or cell-substrate adhesion of particular lineage groups [73]. Reproducible differences in the ratio of average single cell size and cluster surface area, R_RC:IC_, were observed upon substrates relative to control surfaces (Figure 5). Interestingly, no changes in R_N_ were detectable across RPC adhered onto different substrates despite these measured differences. This result suggests R_N_ may be more strongly determined by intrinsic cell properties, e.g., developmental stage, rather than by external stimuli, such as ECM composition. The data reinforce the significance of examining collective cell-biomaterial interactions in the development of transplantable retinal biomaterials, which rely upon ECM concentration, pore size, and surface functionalization that can each impact cell clustering.

### 4.4. Collective RPC Migration

In vitro tests examined the collective chemotactic processes of retinal clusters within controlled microenvironments of FGF, a well-studied chemotactic factor of retinal development [56,57]. RPCs were observed to adhere and migrate collectively as clusters in all fields of the μLane system (Figure 6). Measured values of chemotactic index (CI) approaching 1 illustrated that retinal clusters of different sizes exhibited finely tuned chemotactic migration within different gradient fields of FGF [102]. Furthermore, concentration effects were seen to be significant in conjunction with gradient, as cells migrated in both the highest gradient fields and in fields with the highest FGF concentration. These data reinforce the importance of both the absolute concentration of reagent exposed to transplantable cells as well as its chemical release over time in emerging retinal biomaterials [18,103,104]. Large clusters migrated significantly (*p* < 0.05) further in the largest gradient fields, while smaller clusters exhibited no statistical difference in migration distances. Similarly, average path lengths, or cell distances traveled, of singleton cells were observed not to depend on concentration gradient (Figure 7), and average values of the chemotactic index, CI, measuring less than 0.5 to indicate little to no directional migration [34,52,83].

Lastly, we note that individual cells migrated longer distances than cells migrating collectively in clusters, presumably because movement of the former is independent of the cell-to-cell communication that regulates dynamic movement of the latter [104]. Collective chemotaxis is regulated by both the ligand binding of cell surface receptors and cell-cell adhesion between cells. In *Drosophila*, the E-Cadherin molecule, CadN2, regulates adhesion between retinal cell layers [105,106] as well as overall retinal size [107]. Further, the cadN2 molecule is required for RPC targeting of synaptic targets to the visual centers in the brain [108] and is further implicated in the localization of innexin molecules [109] needed for gap junctional communication between retinal neurons and glia [110]. Although elucidation of these molecules is significant for the collective migration needed in cell replacement therapy, the goal of the current project was to examine the extent to which RPC cluster size impacted directionality. Future study will stain for CadN2, Inx1, and Inx2 as well as measure mRNA expression levels of these proteins. In addition, future tests will genetically manipulate the regulation of these molecules in *Drosophila* eye-brain complexes to examine subsequent differences in RPC cohesion during chemotaxis.

## 5. Conclusions

The current project examined the collective migratory behavior of RPC disassociated from developing eye-brain complexes of *Drosophila* upon microfluidically-constrained substrate surfaces. Results highlighted the impact of cluster size and neuronal:glial composition on collective chemotaxis and suggested that substrate-enhanced clustering may influence the migration of transplanted cells as singletons and/or clusters. Future study will examine cell-cell adhesions that maintain cluster cohesion during chemotaxis and develop microfluidic systems able to examine chemotactic behaviors of cell groups derived from different stages of retinal development. Study of collective migration of transplantable RPCs will, thereby, aid the development of effective biomaterials that promote directional migration of cells towards desired areas.

## Figures and Tables

**Table 1:** Average cell yield per eye-brain complex. Total numbers of cells and cells per brain for dissections and dissociations of n= 15 and n= 30 eye-brain complexes.

Eye-Brain Complexes	Total # of Cells	Average # of Cells per Brain
N = 15	1.4 ± 0.09 × 10^5^	9.2 ± 0.8 × 10^3^
N = 30	2.9 ± 0.5 × 10^5^	9.9 ± 1.7 × 10^3^

## References

[1] Scarpa E., Mayor R. (2016). Collective cell migration in development. J. Cell Biol..

[2] Aman A., Piotrowski T. (2010). Cell migration during morphogenesis. Dev. Biol..

[3] Haeger A., Wolf K., Zegers M.M., Friedl P. (2015). Collective cell migration: Guidance principles and hierarchies. Trends Cell Biol..

[4] Lamba D., Karl M., Reh T. (2008). Neural regeneration and cell replacement: A view from the eye. Cell Stem Cell.

[5] Macabenta F., Stathopoulos A. (2019). Sticking to a plan: Adhesion and signaling control spatial organization of cells within migrating collectives. Curr. Opin. Genet. Dev..

[6] Norden C., Lecaudey V. (2019). Collective cell migration: General themes and new paradigms. Curr. Opin. Genet. Dev..

[7] Devreotes P.N., Bhattacharya S., Edwards M., PIglesias A., Lampert T., Miao Y. (2017). Excitable Signal Transduction Networks in Directed Cell Migration. Annu. Rev. Cell Dev. Biol..

[8] Roca-Cusachs P., Sunyer R., Trepat X. (2013). Mechanical guidance of cell migration: Lessons from chemotaxis. Curr. Opin. Cell Biol..

[9] Shellard A., Mayor R. (2016). Chemotaxis during neural crest migration. Semin. Cell Dev. Biol..

[10] Swaney K.F., Huang C.H., Devreotes P.N. (2010). Eukaryotic chemotaxis: A network of signaling pathways controls motility, directional sensing, and polarity. Annu. Rev. Biophys..

[11] Ridley A.J., Schwartz M.A., Burridge K., Firtel R.A., Ginsberg M.H., Borisy G., Parsons J.T., Horwitz A.R. (2003). Cell migration: Integrating signals from front to back. Science.

[12] Radice G.P. (1980). Locomotion and cell-substratum contacts of Xenopus epidermal cells in vitro and in situ. J. Cell Sci..

[13] Olson H.M., Nechiporuk A.V. (2018). Using Zebrafish to Study Collective Cell Migration in Development and Disease. Front. Cell Dev. Biol..

[14] Yamada K.M., Sixt M. (2019). Mechanisms of 3D cell migration. Nat. Rev. Mol. Cell Biol..

[15] Szabo A., Mayor R. (2018). Mechanisms of Neural Crest Migration. Annu. Rev. Genet..

[16] Rorth P. (2007). Collective guidance of collective cell migration. Trends Cell Biol..

[17] Ho M.T., Teal C.J., Shoichet M.S. (2019). A hyaluronan/methylcellulose-based hydrogel for local cell and biomolecule delivery to the central nervous system. Brain Res. Bull..

[18] Orive G., Santos-Vizcaino E., Pedraz J.L., Hernandez R.M., Ramirez J.E.V., Dolatshahi-Pirouz A., Khademhosseini A., Peppas N.A., Emerich D.F. (2019). 3D cell-laden polymers to release bioactive products in the eye. Prog. Retin. Eye Res..

[19] Hunt N.C., Hallam D., Chichagova V., Steel D.H., Lako M. (2018). The Application of Biomaterials to Tissue Engineering Neural Retina and Retinal Pigment Epithelium. Adv. Healthc. Mater..

[20] Warre-Cornish K., Barber A.C., Sowden J.C., Ali R.R., Pearson R.A. (2014). Migration, integration and maturation of photoreceptor precursors following transplantation in the mouse retina. Stem Cells Dev..

[21] Barber A.C., Hippert C., Duran Y., West E.L., Bainbridge J.W., Warre-Cornish K., Luhmann U.F., Lakowski J., Sowden J.C., Ali R.R. (2013). Repair of the degenerate retina by photoreceptor transplantation. Proc. Natl. Acad. Sci. USA.

[22] Reh T.A. (2016). Photoreceptor Transplantation in Late Stage Retinal Degeneration. Investig. Ophthalmol. Vis. Sci..

[23] Unachukwu U.J., Warren A., Li Z., Mishra S., Zhou J., Sauane M., Lim H., Vazquez M., Redenti S. (2016). Predicted molecular signaling guiding photoreceptor cell migration following transplantation into damaged retina. Sci. Rep..

[24] Sonnen K.F., Merten C.A. (2019). Microfluidics as an Emerging Precision Tool in Developmental Biology. Dev. Cell.

[25] Millet L.J., Gillette M.U. (2012). New perspectives on neuronal development via microfluidic environments. Trends Neurosci..

[26] Liu Z., Han X., Qin L. (2016). Recent Progress of Microfluidics in Translational Applications. Adv. Healthc. Mater..

[27] Sackmann E.K., Fulton A.L., Beebe D.J. (2014). The present and future role of microfluidics in biomedical research. Nature.

[28] Gupta N., Renugopalakrishnan V., Liepmann D., Paulmurugan R., Malhotra B.D. (2019). Cell-based biosensors: Recent trends, challenges and future perspectives. Biosens. Bioelectron..

[29] Lynch M., Ramalingam N. (2019). Integrated Fluidic Circuits for Single-Cell Omics and Multi-omics Applications. Adv. Exp. Med. Biol..

[30] Kaushik G., Leijten J., Khademhosseini A. (2017). Concise Review: Organ Engineering: Design, Technology, and Integration. Stem Cells.

[31] Song M.J., Bharti K. (2016). Looking into the future: Using induced pluripotent stem cells to build two and three dimensional ocular tissue for cell therapy and disease modeling. Brain Res..

[32] Ingber D.E. (2018). Developmentally inspired human ‘organs on chips’. Development.

[33] Thakur A., Mishra S., Pena J., Zhou J., Redenti S., Majeska R., Vazquez M. (2018). Collective adhesion and displacement of retinal progenitor cells upon extracellular matrix substrates of transplantable biomaterials. J. Tissue Eng..

[34] Mishra S., Thakur A., Redenti S., Vazquez M. (2015). A model microfluidics-based system for the human and mouse retina. Biomed. Microdevices.

[35] Karimi M., Bahrami S., Mirshekari H., Basri S.M., Nik A.B., Aref A.R., Akbari M., Hamblin M.R. (2016). Microfluidic systems for stem cell-based neural tissue engineering. Lab Chip.

[36] Uzel S.G., Amadi O.C., Pearl T.M., Lee R.T., So P.T., Kamm R.D. (2016). Simultaneous or Sequential Orthogonal Gradient Formation in a 3D Cell Culture Microfluidic Platform. Small.

[37] Nery F.C., Atai N.A., da Hora C.C., Kim E.Y., Hettich J., Mempel T.R., Breakefield X.O., Irimia D. (2014). Microfluidic platform to evaluate migration of cells from patients with DYT1 dystonia. J. Neurosci. Methods.

[38] Reyes D.R., Perruccio E.M., Becerra S.P., Locascio L.E., Gaitan M. (2004). Micropatterning neuronal cells on polyelectrolyte multilayers. Langmuir.

[39] Fleck O., Savin T. (2018). A physical approach to model occlusions in the retinal microvasculature. Eye Lond..

[40] Terrell D., Comander J. (2019). Current Stem-Cell Approaches for the Treatment of Inherited Retinal Degenerations. Semin. Ophthalmol..

[41] Gagliardi G., M’Barek K.B., Goureau O. (2019). Photoreceptor cell replacement in macular degeneration and retinitis pigmentosa: A pluripotent stem cell-based approach. Prog. Retin. Eye Res..

[42] Narayanan K., Mishra S., Singh S., Pei M., Gulyas B., Padmanabhan P. (2017). Engineering Concepts in Stem Cell Research. Biotechnol. J..

[43] Park J., Baranov P., Aydin A., Abdelgawad H., Singh D., Niu W., Kurisawa M., Spector M., Young M.J. (2019). In Situ Cross-linking Hydrogel as a Vehicle for Retinal Progenitor Cell Transplantation. Cell Transplant..

[44] Singh R., Cuzzani O., Binette F., Sternberg H., West M.D., Nasonkin I.O. (2018). Pluripotent Stem Cells for Retinal Tissue Engineering: Current Status and Future Prospects. Stem Cell Rev. Rep..

[45] Yao J., Ko C.W., Baranov P.Y., Regatieri C.V., Redenti S., Tucker B.A., Mighty J., Tao S.L., Young M.J. (2015). Enhanced differentiation and delivery of mouse retinal progenitor cells using a micropatterned biodegradable thin-film polycaprolactone scaffold. Tissue Eng. Part A.

[46] Stern J.H., Tian Y., Funderburgh J., Pellegrini G., Zhang K., Goldberg J.L., Ali R.R., Young M., Xie Y., Temple S. (2018). Regenerating Eye Tissues to Preserve and Restore Vision. Cell Stem Cell.

[47] Volkner M., Kurth T., Karl M.O. (2019). The Mouse Retinal Organoid Trisection Recipe: Efficient Generation of 3D Retinal Tissue from Mouse Embryonic Stem Cells. Methods Mol. Biol..

[48] Fligor C.M., Langer K.B., Sridhar A., Ren Y., Shields P.K., Edler M.C., Ohlemacher S.K., Sluch V.M., Zack D.J., Zhang C. (2018). Three-Dimensional Retinal Organoids Facilitate the Investigation of Retinal Ganglion Cell Development, Organization and Neurite Outgrowth from Human Pluripotent Stem Cells. Sci. Rep..

[49] Kumar J.P. (2018). The fly eye: Through the looking glass. Dev. Dyn..

[50] Contreras E.G., Sierralta J., Oliva C. (2019). Novel Strategies for the Generation of Neuronal Diversity: Lessons From the Fly Visual System. Front. Mol. Neurosci..

[51] Gaspar P., Almudi I., Nunes M.D.S., McGregor A.P. (2019). Human eye conditions: Insights from the fly eye. Hum. Genet..

[52] Beck C., Singh T., Farooqi A., Venkatesh T., Vazquez M. (2016). Controlled microfluidics to examine growth-factor induced migration of neural progenitors in the Drosophila visual system. J. Neurosci. Methods.

[53] Soderstrom K.O. (1988). Lectin binding to the human retina. Anat. Rec..

[54] Chen M., Harvey A.R., Dyson S.E. (1991). Regrowth of lesioned retinal axons associated with the transplantation of Schwann cells to the brachial region of the rat optic tract. Restor. Neurol. Neurosci..

[55] Guo Y., Wang P., Ma J.H., Cui Z., Yu Q., Liu S., Xue Y., Zhu D., Cao J., Li Z. (2019). Modeling Retinitis Pigmentosa: Retinal Organoids Generated From the iPSCs of a Patient With the USH2A Mutation Show Early Developmental Abnormalities. Front. Cell Neurosci..

[56] Huang P., Stern M.J. (2005). FGF signaling in flies and worms: More and more relevant to vertebrate biology. Cytokine Growth Factor Rev..

[57] Bae Y.K., Trisnadi N., Kadam S., Stathopoulos A. (2012). The role of FGF signaling in guiding coordinate movement of cell groups: Guidance cue and cell adhesion regulator?. Cell Adhes. Migr..

[58] Duffy J.B. (2002). GAL4 system in Drosophila: A fly geneticist’s Swiss army knife. Genesis.

[59] Yamaguchi M., Yoshida H. (2018). Drosophila as a Model Organism. Adv. Exp. Med. Biol..

[60] Lerit D.A., Plevock K.M., Rusan N.M. (2014). Live imaging of Drosophila larval neuroblasts. J. Vis. Exp..

[61] Moraru M.M., Egger B., Bao D.B., Sprecher S.G. (2012). Analysis of cell identity, morphology, apoptosis and mitotic activity in a primary neural cell culture system in Drosophila. Neural Dev..

[62] Wu C.F., Suzuki N., Poo M.M. (1983). Dissociated neurons from normal and mutant Drosophila larval central nervous system in cell culture. J. Neurosci..

[63] Schneider I. (1972). Cell lines derived from late embryonic stages of Drosophila melanogaster. J. Embryol. Exp. Morphol..

[64] Luhur A., Klueg K.M., Zelhof A.C. (2019). Generating and working with Drosophila cell cultures: Current challenges and opportunities. Wiley Interdiscip. Rev. Dev. Biol..

[65] Kong Q., Able R.A., Dudu V., Vazquez M. (2010). A microfluidic device to establish concentration gradients using reagent density differences. J. Biomech. Eng..

[66] Able R.A., Ngnabeuye C., Beck C., Holland E.C., Vazquez M. (2012). Low Concentration Microenvironments Enhance the Migration of Neonatal Cells of Glial Lineage. Cell. Mol Bioeng..

[67] Dudu V., Able R.A., Rotari V., Kong Q., Vazquez M. (2012). Role of Epidermal Growth Factor-Triggered PI3K/Akt Signaling in the Migration of Medulloblastoma-Derived Cells. Cell. Mol. Bioeng..

[68] Rico-Varela J., Singh T., McCutcheon S., Vazquez M. (2015). EGF as a New Therapeutic Target for Medulloblastoma Metastasis. Cell. Mol. Bioeng..

[69] Jones T.A. (1969). Skewness and kurtosis as criteria of normality in observed frequency distributions. J. Sediment. Res..

[70] George D. (2011). SPSS for Windows Step by Step: A Simple Study Guide and Reference, 17.0 Update, 10/e.

[71] Jarque C.M., Bera A.K. (1987). A Test for Normality of Observations and Regression Rezsiduals. International Statistical Review/Revue Internationale de Statistiqu.

[72] Reese B.E. (2011). Development of the retina and optic pathway. Vision Res..

[73] Wu Q., Kumar N., Velagala V., Zartman J.J. (2019). Tools to reverse-engineer multicellular systems: Case studies using the fruit fly. J. Biol. Eng..

[74] Cagan R. (2009). Principles of Drosophila eye differentiation. Curr. Top. Dev. Biol..

[75] Hsiung F., Moses K. (2002). Retinal development in Drosophila: Specifying the first neuron. Hum. Mol. Genet..

[76] Singh A., Irvine K.D. (2012). Drosophila as a model for understanding development and disease. Dev. Dyn..

[77] Jones M.K., Lu B., Girman S., Wang S. (2017). Cell-based therapeutic strategies for replacement and preservation in retinal degenerative diseases. Prog. Retin. Eye Res..

[78] Delgado R., Delgado M.G., Bastin-Heline L., Glavic A., O’Day P.M., Bacigalupo J. (2019). Light-Induced Opening of the TRP Channel in Isolated Membrane Patches Excised from Photosensitive Microvilli from Drosophila Photoreceptors. Neuroscience.

[79] Fan A., Tofangchi A., de Venecia M., Saif T. (2018). A simple microfluidic platform for the partial treatment of insuspendable tissue samples with orientation control. Lab Chip.

[80] Van Giesen L., Neagu-Maier G.L., Kwon J.Y., Sprecher S.G. (2016). A microfluidics-based method for measuring neuronal activity in Drosophila chemosensory neurons. Nat. Protoc..

[81] Mishra B., Ghannad-Rezaie M., Li J., Wang X., Hao Y., Ye B., Chronis N., Collins C.A. (2014). Using microfluidics chips for live imaging and study of injury responses in Drosophila larvae. J. Vis. Exp..

[82] Dolph P., Nair A., Raghu P. (2011). Preparation of dissociated ommatidia from Drosophila. Cold Spring Harb. Protoc..

[83] McCutcheon S., Unachukwu U., Thakur A., Majeska R., Redenti S., Vazquez M. (2017). In vitro formation of neuroclusters in microfluidic devices and cell migration as a function of stromal-derived growth factor 1 gradients. Cell Adhes. Migr..

[84] Tucker B.A., Redenti S.M., Jiang C., Swift J.S., Klassen H.J., Smith M.E., Wnek G.E., Young M.J. (2010). The use of progenitor cell/biodegradable MMP2-PLGA polymer constructs to enhance cellular integration and retinal repopulation. Biomaterials.

[85] Ballios B.G., Cooke M.J., van der Kooy D., Shoichet M.S. (2010). A hydrogel-based stem cell delivery system to treat retinal degenerative diseases. Biomaterials.

[86] Chiba C., Sakai H., Kaneko Y., Saito T. (1995). Concanavalin A Promotes Regeneration of Processes of Isolated Ganglion Cells from the Adult Newt Retina. Zool Sci.

[87] Suzuki T., Mandai M., Akimoto M., Yoshimura N., Takahashi M. (2006). The simultaneous treatment of MMP-2 stimulants in retinal transplantation enhances grafted cell migration into the host retina. Stem Cells.

[88] Erdiakov A.K., Tikhonovich M.V., Rzhavina E.M., Gavrilova S.A. (2015). The Characteristics of Retina at the Development of Proliferative Vitreoretinopathy in Rats after Intraocular Injection of Concanavalin a and Dispase. Ross. Fiziol. Zhurnal Im. IM Sechenova.

[89] Tikhonovich M.V., Erdiakov A.K., Gavrilova S.A. (2018). Nonsteroid anti-inflammatory therapy suppresses the development of proliferative vitreoretinopathy more effectively than a steroid one. Int. Ophthalmol..

[90] Ishikawa M., Watanabe H., Koike Y., Hisatomi O., Tokunaga F., Tonosaki A. (1989). Demonstration by lectin cytochemistry of rod and cone photoreceptors in the lamprey retina. Cell Tissue Res..

[91] Sasaki H., Karasawa K., Hironaka K., Tahara K., Tozuka Y., Takeuchi H. (2013). Retinal drug delivery using eyedrop preparations of poly-L-lysine-modified liposomes. Eur. J. Pharm. Biopharm..

[92] Du J., Sun Y., Li F.H., Du L.F., Duan Y.R. (2017). Enhanced delivery of biodegradable mPEG-PLGA-PLL nanoparticles loading Cy_3_-labelled PDGF-BB siRNA by UTMD to rat retina. J. Biosci..

[93] Tezcaner A., Hicks D., Boulmedais F., Sahel J., Schaaf P., Voegel J.C., Lavalle P. (2006). Polyelectrolyte multilayer films as substrates for photoreceptor cells. Biomacromolecules.

[94] Inatani M., Honjo M., Otori Y., Oohira A., Kido N., Tano Y., Honda Y., Tanihara H. (2001). Inhibitory effects of neurocan and phosphacan on neurite outgrowth from retinal ganglion cells in culture. Investig. Ophthalmol. Vis. Sci..

[95] Varshney S., Hunter D.D., Brunken W.J. (2015). Extracellular Matrix Components Regulate Cellular Polarity and Tissue Structure in the Developing and Mature Retina. J. Ophthalmic Vis. Res..

[96] Assawachananont J., Mandai M., Okamoto S., Yamada C., Eiraku M., Yonemura S., Sasai Y., Takahashi M. (2014). Transplantation of embryonic and induced pluripotent stem cell-derived 3D retinal sheets into retinal degenerative mice. Stem Cell Rep..

[97] Aizawa Y., Shoichet M.S. (2012). The role of endothelial cells in the retinal stem and progenitor cell niche within a 3D engineered hydrogel matrix. Biomaterials.

[98] Sheibani N., Wang S., Darjatmoko S.R., Fisk D.L., Shahi P.K., Pattnaik B.R., Sorenson C.M., Bhowmick R., Volpert O.V., Albert D.M. (2019). Novel anti-angiogenic PEDF-derived small peptides mitigate choroidal neovascularization. Exp. Eye Res..

[99] Napolitano F., di Iorio V., di Iorio G., Melone M.A.B., Gianfrancesco F., Simonelli F., Esposito T., Testa F., Sampaolo S. (2019). Early posterior vitreous detachment is associated with LAMA5 dominant mutation. Ophthalmic Genet..

[100] Shen D., Wen R., Tuo J., Bojanowski C.M., Chan C.C. (2006). Exacerbation of retinal degeneration and choroidal neovascularization induced by subretinal injection of Matrigel in CCL2/MCP-1-deficient mice. Ophthalmic Res..

[101] Camley B.A., Zimmermann J., Levine H., Rappel W.J. (2016). Collective Signal Processing in Cluster Chemotaxis: Roles of Adaptation, Amplification, and Co-attraction in Collective Guidance. PLoS Comput. Biol..

[102] Delplace V., Ortin-Martinez A., Tsai E.L.S., Amin A.N., Wallace V., Shoichet M.S. (2019). Controlled release strategy designed for intravitreal protein delivery to the retina. J. Control. Release.

[103] Wong F.S., Wong C.C., Chan B.P., Lo A.C. (2016). Sustained Delivery of Bioactive GDNF from Collagen and Alginate-Based Cell-Encapsulating Gel Promoted Photoreceptor Survival in an Inherited Retinal Degeneration Model. PLoS ONE.

[104] Ellison D., Mugler A., Brennan M.D., Lee S.H., Huebner R.J., Shamir E.R., Woo L.A., Kim J., Amar P., Nemenman I. (2016). Cell-cell communication enhances the capacity of cell ensembles to sense shallow gradients during morphogenesis. Proc. Natl. Acad. Sci. USA.

[105] Dumstrei K., Wang F., Hartenstein V. (2003). Role of DE-cadherin in neuroblast proliferation, neural morphogenesis, and axon tract formation in Drosophila larval brain development. J. Neurosci..

[106] Hill E., Broadbent I.D., Chothia C., Pettitt J. (2001). Cadherin superfamily proteins in Caenorhabditis elegans and Drosophila melanogaster. J. Mol. Biol..

[107] Richard M., Hoch M. (2015). Drosophila eye size is determined by Innexin 2-dependent Decapentaplegic signalling. Dev. Biol..

[108] Lee C.H., Herman T., Clandinin T.R., Lee R., Zipursky S.L. (2001). N-cadherin regulates target specificity in the Drosophila visual system. Neuron.

[109] Bauer R., Weimbs A., Lechner H., Hoch M. (2006). DE-cadherin, a core component of the adherens junction complex modifies subcellular localization of the Drosophila gap junction protein innexin2. Cell Commun. Adhes..

[110] Welzel G., Schuster S. (2018). Long-term potentiation in an innexin-based electrical synapse. Sci. Rep..

